# Leg Locomotion Adaption for Quadruped Robots with Ground Compliance Estimation

**DOI:** 10.1155/2020/8854411

**Published:** 2020-09-21

**Authors:** Songyuan Zhang, Hongji Zhang, Yili Fu

**Affiliations:** State Key Laboratory of Robotics and System, Harbin Institute of Technology, 150001, China

## Abstract

Locomotion control for quadruped robots is commonly applied on rigid terrains with modelled contact dynamics. However, the robot traversing different terrains is more important for real application. In this paper, a single-leg prototype and a test platform are built. The Cartesian coordinates of the foot-end are obtained through trajectory planning, and then, the virtual polar coordinates in the impedance control are obtained through geometric transformation. The deviation from the planned and actual virtual polar coordinates and the expected force recognized by the ground compliance identification system are sent to the impedance controller for different compliances. At last, several experiments are carried out for evaluating the performance including the ground compliance identification, the foot-end trajectory control, and the comparison between pure position control and impedance control.

## 1. Introduction

Currently, the main forms of locomotion robots include legged robots and tracked/wheeled robots. Compared with a tracked or wheeled robot, legged robots can easily adapt to and walk on rough terrain [1]. Legged robots can choose contact points with environment for overcoming obstacles and finding the feasible stable region [2]. For example, Boston Dynamics developed a hydraulic driven BigDog robot, which aimed at building an unmanned legged robot with rough-terrain mobility [3]. MIT Cheetah was designed with proprioceptive actuator for impact mitigation and high-bandwidth physical interaction [4]. In our previous research, inspired by the proprioceptive actuator [5], we designed a single-leg platform for high-speed locomotion. ANYmal used the complicated actuator which makes the robot impact with high torque control accuracy [6]. Overall, the hydraulic actuator has the feature of naturally robust against impulsive loads with a high-power density [7, 8]. However, legged robots with hydraulic actuators such as Bigdog developed by Boston Dynamics are difficult to be scaled down and will generate large noise [9]. Different from that, the electric actuator with proprioceptive design allows the force proprioception which can deliver the desired force with motor current sensing [10].

For quadruped robot to achieve better performance, foot trajectory planning is crucial. Two factors should be considered in the design of the foot trajectory. One is that the great impact should be avoided when the feet land on the ground; another is that the leg structure should have enough ground clearance for avoiding obstacles [11]. The foot trajectory also requires continuous velocity and acceleration for stability. There are three main methods to plan the trajectory of a quadruped robot, i.e., Bézier curve trajectory, cubic trajectory, and sinusoidal trajectory [11]. Among them, the Bézier curve meets the above requirements perfectly. After the foot trajectory planning, the behaviour that the robot interacts with irregular ground should be considered. Quadruped robots which can adjust the foot-end dynamic behaviour to deal with the unstructured terrains are important on real application. For example, Semini et al. implemented an impedance controller to control the electrohydraulically driven leg of HyQ [12]. Hyun et al. realized the virtual leg compliance of the MIT Cheetah with proprioceptive impedance control to deal with the external disturbance. After knowing the coordinates in the Cartesian coordinate system, the joint coordinates are obtained by inverse kinematic transformation, and the joint motion trajectory is obtained. Moreover, the whole leg movement of quadruped robots will be divided into stance phase with impedance control and swing phase with position control [13]. However, rigid ground assumption is applied for most researches. The different contact dynamics between the rigid assumption and soft contact will affect the performance and stability of robots. In contrast to that, Kim et al. penalizing the contact interaction in the cost function during the design of the whole-body controller [14]. Neunert et al. used the soft contact model combined with MPC controller [15]. Bosworth et al. designed a controller which can be tuned for different ground types [16]. Different from that, in our study, the least-squares method is applied to estimate the ground compliance parameters. The method is a soft terrain adaption algorithm which can achieve a transition between hard and soft ground with a real-time terrain-aware. For real leg locomotion adaption, the estimated stiffness of ground will be used for adjusting the parameters of impedances controller.

This paper is structured as follows: Section 2 introduces the kinematic modelling method for the leg. In Section 2, the foot trajectory of the leg is designed utilizing the Bézier curve and sinusoidal waves. The detailed implementation of impedance control and ground compliance identification will be introduced in Section 2. The experimental results are provided in Section 3. At last, the conclusions are given.

## 2. Materials and Methods

**Figure 1:**
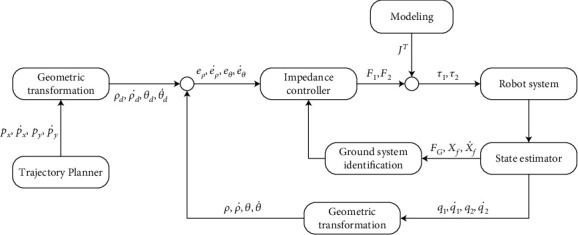
Overall of the control diagram.

The detailed leg design with three-joint leg structure can be found in our previous research [5]. The overall of the control diagram is shown in Figure 1. During the movement of robots, the Cartesian coordinates of the foot-end are obtained through trajectory planning, and then, the virtual polar coordinates in the impedance control are obtained through geometric transformation. The deviation from the planned and actual virtual polar coordinates and the expected force recognized by the ground identification system is sent to the impedance controller. Finally, the desired foot-end force is obtained and then, the joint torque can be calculated by the change of the Jacobian matrix and sent to the robot system. The status of the robot system is also fed back to the previous process.

### 2.1. One Leg Kinematic Analysis

**Figure 2:**
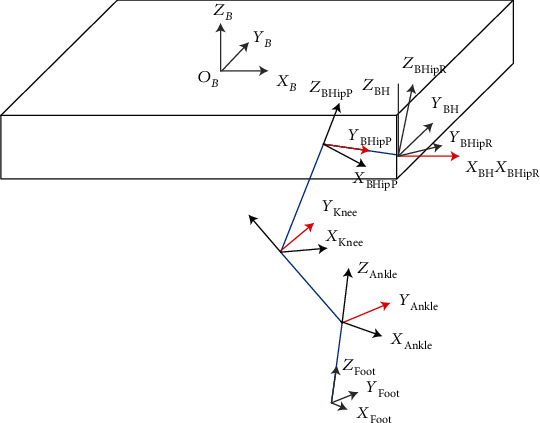
Link coordinate systems for the right front leg of the quadruped robot.

For realizing the foot trajectory control of the leg, the kinematics formula should be derived first. To simplify the subsequent analysis of the motion control problems, the first step is to establish a complete coordinate system for a quadruped robot. Since the configuration of the four legs of the robot is identical, the only difference is the position of the hip joint relative to the centre of mass (COM) of the robot. Therefore, the kinematics analysis of the foot-end with the hip coordinate system is consistent for all four legs. Here, only the right front leg is selected for the analysis in this section. The following joint coordinate systems are established according to the Denavit-Hartenberg (D-H) method: the hip rolling coordinate system ∑_HipR_, the hip pitch coordinate system ∑_HipP_, the knee pitch coordinate system ∑_Knee_, the ankle pitch coordinate system ∑_Ankle_, and the foot-end coordinate system ∑_Foot_ are shown in Figure 2. The blue line in the figure shows the three links of the leg; the red line shows the axes of the leg rotating joints.

**Figure 3:**
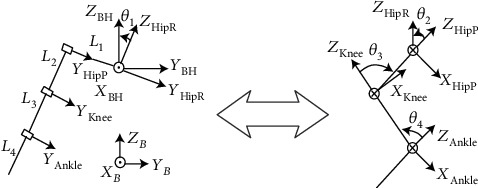
One leg kinematic model of the quadruped robot.

The coordinate system of each link, as well as four joint angles, is shown in Figure 3 in detail. The left picture is the front view, where the *X*_BH_ axis of the hip torso coordinate system is perpendicular to the paper surface. The right picture is a schematic plan view of the right front leg, the rotation axes of the hip pitch, knee pitch, and ankle pitch joints which are oriented perpendicular to the paper in. In addition, according to the right-hand rule, from the foot-end to the hip joint, the ankle pitch joint *θ*_4_, knee pitch joint *θ*_3_, hip pitch joint *θ*_2_, and hip roll joint *θ*_1_ are defined.

According to the given kinematic model of the quadruped robot, from the hip pitch coordinate system ∑_BH_ to the foot-end coordinate system ∑_Foot_, the homogeneous transformation matrix between the neighbouring link coordinate systems can be derived. Firstly, the homogeneous transformation matrix of the hip rolling coordinate system relative to the hip torso coordinate system is

(1) THipRBH=10000cos−θ1−sin−θ100sin−θ1cos−θ100001.

The homogeneous transformation matrix of the hip pitch coordinate system relative to the hip rolling coordinate system is

(2) THipPHipR=cosθ20sinθ20010−L1−sinθ20cosθ200001.

The homogeneous transformation matrix of the knee pitch coordinate system relative to the hip pitch coordinate system is

(3) TKneeHipP=cos−θ30sin−θ300100−sin−θ30cos−θ3−L20001.

The homogeneous transformation matrix of the ankle pitch coordinate system relative to the knee pitch coordinate system is

(4) TAnkleKnee=cosθ40sinθ400100−sinθ40cosθ4−L30001,

The homogeneous transformation matrix of the foot-end coordinate system relative to the ankle pitch coordinate system is

(5) TFootAnkle=10000100001−L40001.

At last, by integrating these transformation matrixes, the (6) and (7) can be derived

(6) TFootBH=THipRBH·THipPHipR·TKneeHipP·TAnkleKnee·TFootAnkle,

(7) TFootBH=c2−3+40s2−3+4−L4s2−3+4−L3s2−3−L2s2−s1s2−3+4c1s1c2−3+4−L1c1−L2c2+L3c2−3+L4c2−3+4s1−c1s2−3+4−s1c1c2−3+4L1s1−L2c2+L3c2−3+L4c2−3+4c10001,

where *c*_1_ represents cos*θ*_1_, *s*_1_ represents sin*θ*_1_, *c*_2_ represents cos*θ*_2_,*c*_2−3_ represents cos(*θ*_2_ − *θ*_3_),*s*_2−3_ represents sin(*θ*_2_ − *θ*_3_),*c*_2−3+4_ represents cos(*θ*_2_ − *θ*_3_ + *θ*_4_), and *s*_2−3+4_ represents sin(*θ*_2_ − *θ*_3_ + *θ*_4_).

**Figure 4:**
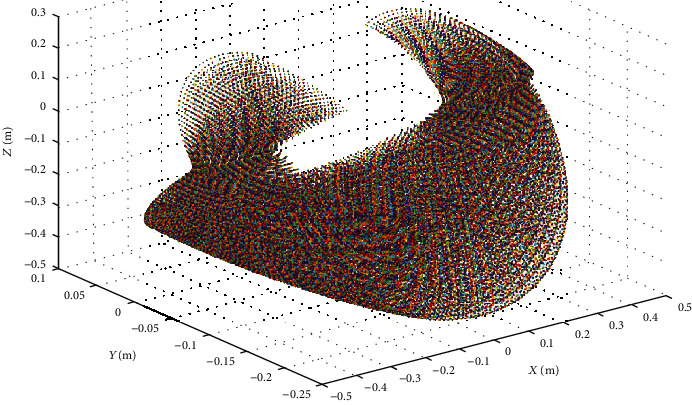
3D workspace point map of one leg.

**Figure 5:**
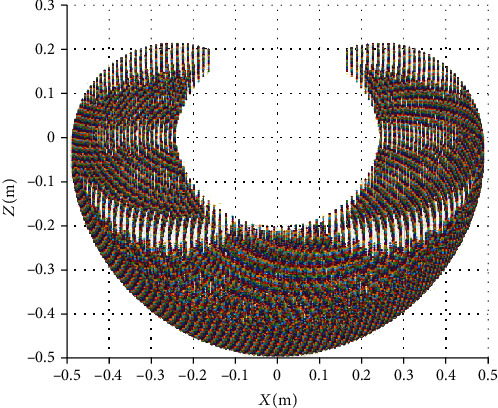
*X*_HipR_ *Z*_HipR_-plane workspace point map.

Therefore, according to the positive kinematic analysis, in the case of the known joint angle *θ*_1_, *θ*_2_, *θ*_3_, *θ*_4_, the position and orientation of the foot-end relative to the hip torso coordinate system can be obtained. For calculating the inverse solution, the solvability should be considered for avoiding the no solution or multiple solutions. Usually, solving robot kinematic equations by the inverse operation is a nonlinear problem, and solving forward kinematic problems is to check whether the target point is in the working space. Therefore, for deciding the existence of the robot inverse kinematics solution, the robot leg's workspace should be calculated. For ensuring that the inverse kinematic is solvable, the foot of the quadruped robot must be within the workspace of the leg joint. According to the design index *θ*_1_ ∈ [−20^o^, 20^o^],*θ*_2_ ∈ [−50^o^, 50^o^], *θ*_3_ ∈ [−120^o^, 120^o^], a series of coordinate points can be obtained with different joint angles. A point cloud map indicting the whole working space of the leg is shown in Figures 4 and 5.

For the solution of inverse kinematic problems, there are mainly two types of closed-form solutions (analytic solutions) and numerical solutions. In this paper, the closed solution method is used to solve the analytical solution. Because the quadruped robot has the same leg configuration, only the right front leg is considered to establish its inverse kinematics model. From equation (7), we can get

(8) PBHx2+PBHy2+PBHz2=L12+L22+L32+L42+2L2L3cosθ3+2L3L4cosθ4+2L2L4cosθ3−θ4.

Because of the parallelogram structure, it is structurally guaranteed that the (8) simplifies to

(9) PBHx2+PBHy2+PBHz2=L12+L22+L32+L42+2L2L3+L3L4cosθ3+2L2L4.

So, we can obtain

(10) θ3=θ4=arccosPBHx2+PBHy2+PBHz2−L12−L22−L32−L42−2L2L42L2L3+L3L4.

From equation (7), we get

(11) PBHy+L1cosθ1PBHz+L1sinθ1=sinθ1cosθ1.

Further,

(12) −PBHzPBHy2+PBHz2sinθ1−−PBHyPBHy2+PBHz2cosθ1=−L1PBHy2+PBHz2.

So,

(13) θ1=arcsin−L1PBHy2+PBHz2+arctanPBHyPBHz.

From equation (7), we can also get

(14) PBHx=−L4+L2+L3cosθ3sinθ2+L3sinθ3cosθ2.

Since *θ*_3_ has been given by equation (10), so

(15) θ2=arcsinPBHxL4+L2+L3cosθ32+L3sinθ32+arctanL3sinθ3L4+L2+L3cosθ3.

Formula (10), formula (13), and formula (15) are the inverse kinematic equations of the leg, and the inverse solution is the only solution. Thus, if we know the coordinates of the foot-end of any leg in the hip joint body coordinate system, we can solve the requirement of each joint angle.

### 2.2. Velocity Jacobian Matrix

In this section, the velocity Jacobian matrix is derived which is useful for further leg control such as the transformation of forces and torques from the foot-end to the joints. The definition of the Jacobian matrix is as follows:

(16) J=∂PBHx∂θ1∂PBHx∂θ2∂PBHx∂θ3∂PBHy∂θ1∂PBHy∂θ2∂PBHy∂θ3∂PBHz∂θ1∂PBHz∂θ2∂PBHz∂θ3.

Then, according to the equation of positive kinematics, the Jacobian matrix of the velocity from the leg joint coordinates to the foot can be obtained as

(17) $$\begin{array}{c} J=\left[\begin{array}{ccc} 0 & l_{2}c_{2}+l_{3}c_{23} & l_{3}c_{23}\\ l_{1}s_{1}+l_{2}c_{1}c_{2}+l_{3}c_{1}c_{23} & -s_{1}\left(l_{3}s_{23}+l_{2}s_{2}\right) & -l_{3}s_{1}s_{23}\\ -l_{1}c_{1}+l_{2}s_{1}c_{2}+l_{3}s_{1}c_{23} & c_{1}\left(l_{3}s_{23}+l_{2}s_{2}\right) & l_{3}c_{1}s_{23} \end{array}\right]. \end{array}$$

### 2.3. Foot-End Trajectory Planning

For obtaining better performance of the robot, we should design the foot-end trajectory properly. During the swing phase, the leg is not affected by contact force, so a higher speed and acceleration can be achieved. During the stance phase, the extensive loadings will be added on the legs; it will cause a great rigid impact with only position control. Since the swing phase and the stance phase have different dynamic characteristics, the trajectories of the two phases are designed individually.

Later, for tracking the trajectory, two control methods will be compared which are position control and impedance control.

The swing-phase trajectories are designed from a Bézier curve defined by twelve control points, and stance-phase trajectories are designed as part of sinusoidal wave which has a good performance in smoothness and was also used in other robots [17].

#### 2.3.1. Trajectory Design for the Swing Phase

**Figure 6:**
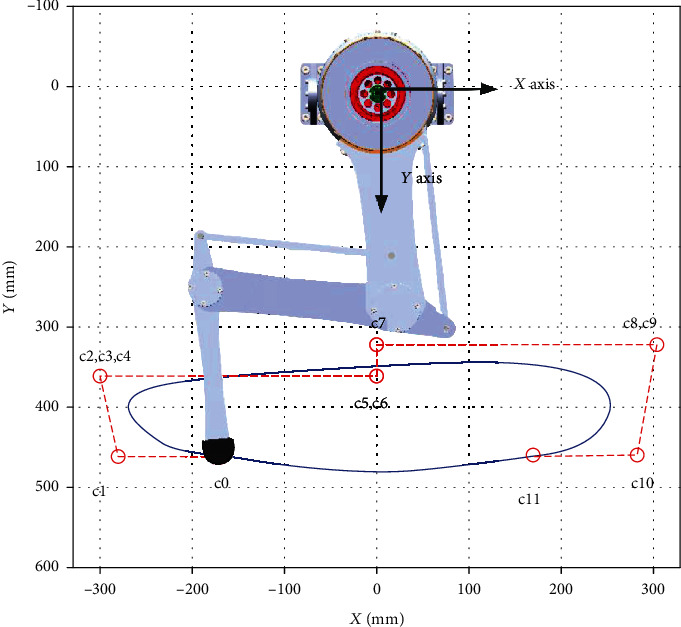
The desired trajectory decided by 12 control points of the Bézier curve.

The swing-phase trajectory design should guarantee enough ground clearance for avoiding obstacles and reduce energy losses during touchdown motion [18]. The design of the swing-phase trajectory should not only approximate the natural behaviour of the leg but also satisfy ground clearance, which can avoid obstacles in the swing phase. The Bézier curve formula determined by the normalization parameter *S*^*SW*^(*t*) ∈ [0, 1] is

(18) $$\begin{array}{c} p^{\text{sw}}\left(t\right)=p^{\text{sw}}\left(S^{\text{sw}}\left(t\right)\right)=\overset{n}{\underset{k=0}{\sum }}C_{n}^{k}{\left(1-S^{\text{sw}}\right)}^{n-k}{\left(S^{\text{sw}}\right)}^{k}c_{k},\\ v^{\text{sw}}t=\frac{{dp}^{\text{sw}}}{{dS}^{\text{sw}}}\frac{{dS}^{sw}}{dt}=\frac{{dp}^{\text{sw}}}{{dS}^{\text{sw}}}\frac{1}{T_{\text{sw}}}, \end{array}$$

where *C*_*n*_^*k*^ represents the number of unordered collection in which *k* elements are taken from *n* elements, (*n* + 1) is the number of control points, *c*_*k*_ is a *k*th two-dimensional control point where *k* ∈ {0, ⋯⋯, 11}. The curve can be generated by twelve control points as shown in Table 1, and the trajectory shape is shown in Figure 6.

#### 2.3.2. Trajectory Design for the Stance Phase

The stance-phase control of each leg will affect the performance of quadruped locomotion via interaction with the ground. Therefore, the planned trajectory should not only consider the motion requirements but also the interactive force requirements.

The stance-phase trajectory is proposed to simply as a sinusoidal wave with two parameters: the half of the stroke length *L*_span_, and the amplitude variable *δ*. As with the swing phase, the stance-phase trajectory equation is also determined by the normalized parameters, *S*^st^∈[0,1]

(19) $$\begin{array}{c} p_{x}^{\text{st}}\left(t\right)=L_{\text{span}}\left(1-2S^{\text{st}}\left(t\right)\right)+P_{0,x},\\ p_{y}^{\text{st}}\left(t\right)=\delta \cos\left(\frac{\pi }{2L_{\text{span}}}p_{x}^{\text{st}}\left(t\right)\right)+P_{0,y},\\ v_{x}^{\text{st}}\left(t\right)=\frac{{dp}_{x}^{\text{st}}}{{dS}^{\text{st}}}\frac{{dS}^{\text{st}}}{dt}=-\frac{2L_{\text{span}}}{T_{\text{st}}},\\ v_{y}^{\text{st}}\left(t\right)=\frac{{dp}_{y}^{\text{st}}}{{dp}_{x}^{\text{st}}}\frac{{dp}_{x}^{\text{st}}}{dt}=\frac{\delta \pi }{T_{\text{st}}}\sin\left(\frac{\pi }{2L_{\text{span}}}p_{x}^{\text{st}}\left(t\right)\right). \end{array}$$

Considering that the robot's leg will touch the ground and bear an impact, therefore, an impedance control should be used to resist the impact during the stance phase and will be introduced in the next part.

### 2.4. Impedance Controller Design

In the previous section, we discussed the planning for robot's foot-end trajectory. If the robot has no contact with the external environment, pure motion control is enough for trajectory tracking. However, quadruped robots are high dynamic robots that their feet will contact the ground frequently during the movement. In this case, the space constraint brought by the environment will hinder the tracking movement of the robot end effector. Therefore, for ensuring the compliance during the movement, the impedance control is used where the leg will be imitating mass, spring, and damper properties. Based on this, the robot will present virtual mass, stiffness, and damping characteristics during movement [4].

**Figure 7:**
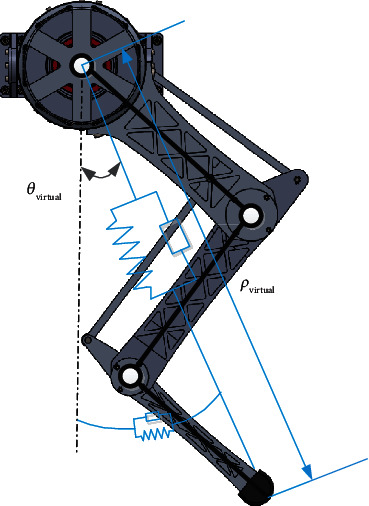
Virtual impedance model of the leg.

A schematic diagram of a one-dimensional mass-damping-spring model is shown in Figure 7. Impedance is used to describe the behavior of a robot. Different impedance parameters can be set to give different dynamic characteristics of the robot.

The dynamics for a one-dof robot rendering an impedance can be written

(20) $$\begin{array}{c} m\ddot{x}+b\dot{x}+kx=f, \end{array}$$

where *x* is the position, *m* is the mass, *b* is the damping, *k* is the stiffness, and *f* is the force applied by the user. If *b* or *k* in the impedance coefficient is set to be large, it is called high impedance; if *b* or *k* is set small, it becomes low impedance. In this paper, virtual leg impedance is created in the polar coordinate as shown in Figure 7.

The control formula can be derived as

(21) $$\begin{array}{c} f_{\text{control}}-f_{\text{est}}=m\left({\ddot{x}}_{d}-\ddot{x}\right)+b\left({\dot{x}}_{d}-\dot{x}\right)+k\left(x_{d}-x\right), \end{array}$$

where *f*_control_ is the control force sent to the controller; *f*_est_ is the estimated ground reaction force; ${\ddot{x}}_{d},{\dot{x}}_{d},x_{d}$ are desired acceleration, velocity, and position; and $\ddot{x},\dot{x},x$ are actual acceleration, velocity, and position. Considering that the virtual mass has no significant effect on the impedance effect of the robot's legs, no virtual mass term is added to the control algorithm in this paper.

The Jacobian from the hip/shoulder to foot-end in the polar coordinate system is obtained by the transformation. The position relationship between the Cartesian coordinate system and the polar coordinate system is

(22) $$\begin{array}{c} \left[\begin{array}{c} \rho_{\text{virtual}}\\ \theta_{\text{virtual}} \end{array}\right]=\left[\begin{array}{c} \sqrt{x^{2}+y^{2}}\\ \arctan\left(x/y\right) \end{array}\right]. \end{array}$$

So the Jacobian from the Cartesian coordinate system to the polar coordinate system is

(23) $$\begin{array}{c} J_{\text{polar}}\left(x,y\right)=\left[\begin{array}{cc} \frac{x}{\sqrt{x^{2}+y^{2}}} & \frac{y}{\sqrt{x^{2}+y^{2}}}\\ \frac{1}{y\left(x^{2}/y^{2}+1\right)} & \frac{-x}{y^{2}\left(x^{2}/y^{2}+1\right)} \end{array}\right]. \end{array}$$

**Figure 8:**
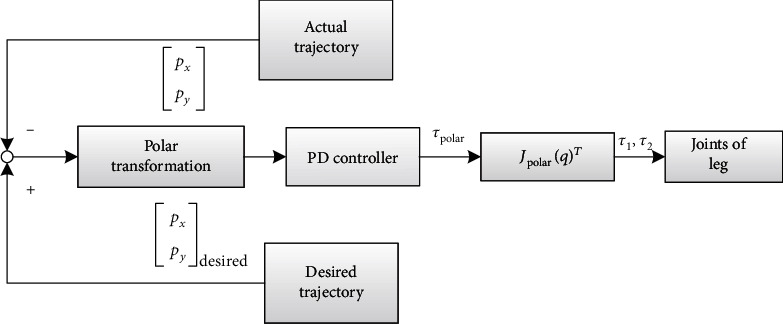
Block diagram for impedance controller.

Then, impedance control law can be derived as

(24) $$\begin{array}{c} \left[\begin{array}{c} \tau_{1}\\ \tau_{2} \end{array}\right]=J_{\text{polar}}{\left(q\right)}^{T}\left[\begin{array}{c} k_{\rho }e_{\rho }+b_{\rho }{\dot{e}}_{\rho }\\ k_{\theta }e_{\theta }+b_{\theta }{\dot{e}}_{\theta } \end{array}\right], \end{array}$$

where $e_{\rho },{\dot{e}}_{\rho },e_{\theta },\text{and }{\dot{e}}_{\theta }$ are radial position error, radial velocity error, angular position error, and angular velocity error between the actual trajectory with designed trajectory, respectively. *J*_polar_(*q*) is the Jacobian from hip/shoulder to foot-end in the polar coordinate system, and *J*_polar_(*q*) = *J*_polar_(*x*, *y*)*J*_Cartesian_(*q*). And the block diagram for the impedance controller can be shown in Figure 8.

### 2.5. Ground Compliance Estimation

For the above analysis, we assumed that the ground is completely rigid, but for a real application, the assumption is limited. For example, if the robot is moving on a concrete floor or an asphalt road, we can think that the assumption is completely valid. But when the robot moves in an environment like grass, marshes, and snow, there will be a big difference. Therefore, the identification of ground compliance is important. Through the system identification method, we can get the stiffness and damping characteristics of the contact ground, and then, further operations to achieve the corresponding impedance characteristics can be implemented.

Among the system identification theory, the least-squares method is widely used, and the effect is also excellent. Therefore, we use the least-squares method to estimate the ground compliance parameters.

Taking into account the storage performance and computational performance of industrial controllers, we estimate the ground parameters using the least squares method in limited memory, which limit the estimated range. The ground reaction force (GRF) is described as

(25) $$\begin{array}{c} f_{G}=k_{G}x_{f}+b_{G}{\dot{x}}_{f}, \end{array}$$

where *k*_*G*_ and *b*_*G*_ are ground stiffness and damping coefficients; that is, the values we need to identify by the system identification *x*_*f*_ and *f*_*G*_ are the depth and the ground reaction force. Then, we can estimate the ground parameters by the least squares method in limited memory.

At every time instant *n*, we gather samples from the previous *k* time instances and compute the ground parameters. By choosing the appropriate *k* value, i.e., the defined range, a good parameter estimate can be obtained and the data saturation phenomenon can be effectively improved.

To implement the estimation of parameters, we construct the following dataset

(26) $$\begin{array}{c} F_{G}={\left[f_{G}\left(n\right)\;f_{G}\left(n-1\right)\cdots f_{G}\left(n-k\right)\right]}^{T},\\ X_{f}={\left[x_{f}\left(n\right)x_{f}\left(n-1\right)\cdots x_{f}\left(n-k\right)\right]}^{T},\\ {\dot{X}}_{f}={\left[{\dot{x}}_{f}\left(n\right){\dot{x}}_{f}\left(n-1\right)\cdots {\dot{x}}_{f}\left(n-k\right)\right]}^{T}. \end{array}$$

By estimating $\hat{\theta }$ and ${\hat{F}}_{G}$ as the optimal estimate of *θ* = [*k*_*G*_ *b*_*G*_]^*T*^ and *F*_*G*_, we can get

(27) $$\begin{array}{c} {\hat{F}}_{G}=\left[\begin{array}{cc} X_{f} & {\dot{X}}_{f} \end{array}\right]\hat{\theta }. \end{array}$$

**Figure 9:**
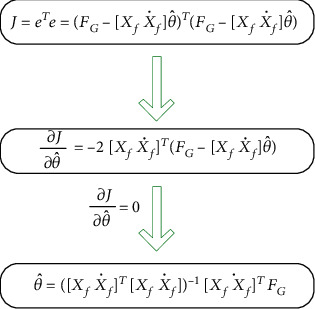
Derivation block diagram of the least-squares method.

Then, by defining $e=F_{G}-{\hat{F}}_{G}$ and *J* = *e*^*T*^*e*, and from that, the least-squares estimation requires that the sum of the squares of the residuals be the smallest; we can obtain the parameters as shown in Figure 9.

Moreover, considering that recent samples make more great impact on the results, we use a weighting matrix *Q* ∈ ℝ^*k*×*k*^ to penalize the error on most recent sample compared to the less recent ones and thus, giving more importance to the most recent samples. And the result is

(28) $$\begin{array}{c} \hat{\theta }={\left({\left[\begin{array}{cc} X_{f} & {\dot{X}}_{f} \end{array}\right]}^{T}Q\left[\begin{array}{cc} X_{f} & {\dot{X}}_{f} \end{array}\right]\right)}^{-1}{\left[\begin{array}{cc} X_{f} & {\dot{X}}_{f} \end{array}\right]}^{T}F_{G}. \end{array}$$

According to the estimates $\hat{\theta }={\left[\begin{array}{cc} {\overset{\wedge }{k}}_{G} & {\overset{\wedge }{b}}_{G} \end{array}\right]}^{T}$, we can acquire

(29) $$\begin{array}{c} {\hat{f}}_{G}={\hat{k}}_{G}x_{f}+{\hat{b}}_{G}{\dot{x}}_{f}. \end{array}$$

Then, in the stance phase, for getting better impedance characteristics for the robot, we apply this force to the impedance control formula (21), and we can get

(30) $$\begin{array}{c} f_{\text{control}}-{\hat{f}}_{G}=m\left({\ddot{x}}_{d}-\ddot{x}\right)+b\left({\dot{x}}_{d}-\dot{x}\right)+k\left(x_{d}-x\right). \end{array}$$

By the least-squares estimation method with limited memory and fading memory for ground parameters, we reasonably introduced the estimated value of GRF. Applying the estimated GRF to the *f*_desired_ impedance controller, the value will change with different ground conditions, allowing the robot to adapt to different ground conditions with suitable compliance.

## 3. Experimental Results

### 3.1. Foot-End Trajectory Experiment

For ensuring that the robot can well perform the planned motion characteristics, an experiment was carried out to verify the robot's performance of foot-end trajectory tracking.

**Figure 10:**
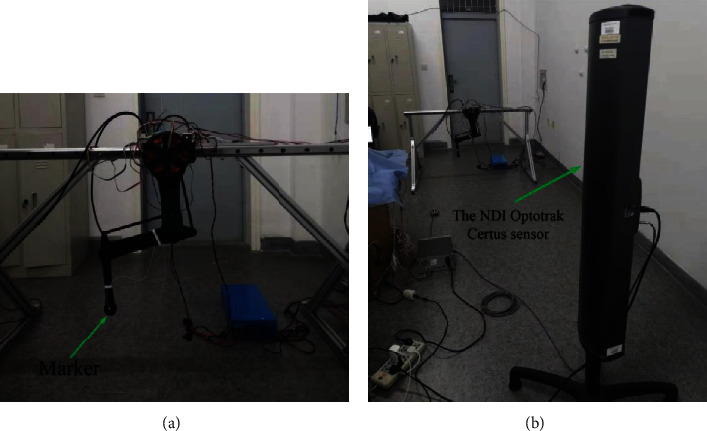
Experimental platform. (a) One leg experimental platform. (b) Trajectory measurement with the NDI Optotrak Certus sensor.

The experimental platform was constructed as shown in Figure 10. The NDI Optotrak Certus was used to trace the presticked marker on the endpoint of the leg, and the actual trajectories can be obtained. The accuracy that NDI Optotrak Certus can achieve is 0.01 mm and its sampling frequency is 100 Hz, which is sufficient for our trajectory following the acquisition.

Because the data collected by the NDI Optotrak Certus is three-dimensional coordinates of a point on its body, and the trajectory points we plan are the two-dimensional coordinates with the axis of the hip joint motor as the origin; it is necessary to compare the trajectories after the coordinate transformation.

The first step is to find the homogeneous transformation matrix ^*O*^*T*_*A*_ from the NDI Optotrak Certus coordinate system *A* to the robot coordinate system *O*.

In the coordinate system *O*, four scattered coordinate points were selected that the foot-end of the robot can reach. Suppose the leg motion is strictly in a plane, and *z* = 0, then assume that the coordinates of the selected four points are (^*O*^*x*_1_, ^*O*^*y*_1_, ^*O*^*z*_1_), (^*O*^*x*_2_, ^*O*^*y*_2_, ^*O*^*z*_2_), (^*O*^*x*_3_, ^*O*^*y*_3_, ^*O*^*z*_3_), and (^*O*^*x*_4_, ^*O*^*y*_4_, ^*O*^*z*_4_).

Simultaneously, the four points are collected by the NDI Optotrak Certus, and the coordinates under frame *A* are (^*A*^*x*_1_, ^*A*^*y*_1_, ^*A*^*z*_1_), (^*A*^*x*_2_, ^*A*^*y*_2_, ^*A*^*z*_2_), (^*A*^*x*_3_, ^*A*^*y*_3_, ^*A*^*z*_3_), and (^*A*^*x*_4_, ^*A*^*y*_4_, ^*A*^*z*_4_).

So

(31) xO1yO1zO11=TOAxA1yA1zA11.

After making the same transformation of all four coordinates and transforming the final matrix equation appropriately, we can get

(32) TOA=xO1xO2xO3xO4yO1yO2yO3yO4zO1zO2zO3zO41111·xA1xA2xA3xA4yA1yA2yA3yA4zA1zA2zA3zA41111−1.

After finding the homogeneous transformation matrix ^*O*^*T*_*A*_, the points collected by the NDI Optotrak Certus are homogeneously transformed to obtain the actual trajectory in the *O* coordinate system and compared with the planned trajectory.

### 3.2. Impedance Control Experiment

For the experiment, the impedance control is based on the control in the torque mode. Therefore, the controller should first be switched to the torque mode at first. In our study, the controller (C6015, Beckhoff) was chosen with EtherCAT connection to each motor.

**Figure 11:**
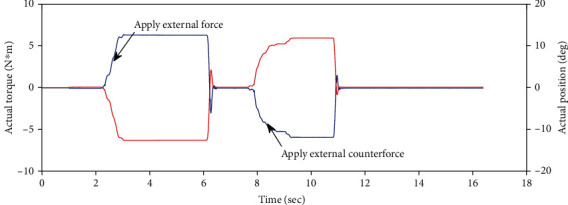
The actual torque and position of the hip joint change after applying force.

**Figure 12:**
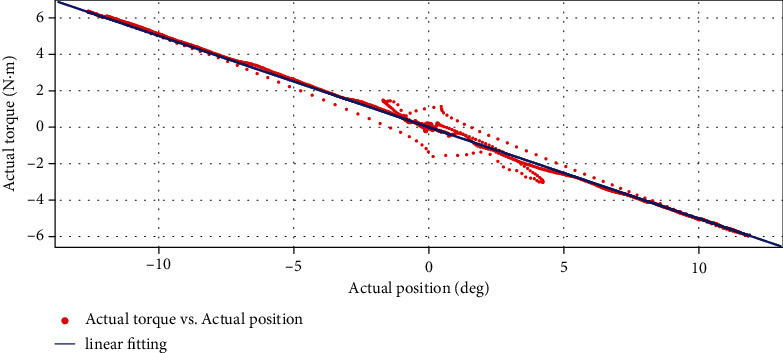
The actual torque change with the position of the hip joint and the fitting straight line.

We observed the relationship between the real-time position, the real-time torque, and time by applying external torque to the hip joint, as shown in Figure 11. Then, we fit the torque position and get the result as shown in Figure 12.

And the fitting equation is

(33) $$\begin{array}{c} y=-0.501x-0.0003333. \end{array}$$

### 3.3. Comparison between Pure Position Control and Impedance Control

As we know, the essence of impedance control is to control the dynamic relationship between force and position. The pure position control is mainly to achieve an accurate position and does not consider the interaction with the outside world. To better understand impedance control and pure position control, we have done experiments to compare the characteristics of the two control methods. According to the characteristics of the two control methods, we can determine which way is more suitable in the locomotion of the quadruped robot.

In the previous analysis, we have also seen that in the swing phase, since the foot-end point of the single-leg prototype has no contact with the ground, the effects of impedance control and pure position control are similar. But in the stance phase, the foot-end point of the single-leg prototype contacts the ground, and there will be a large impact at the moment of contact. If the control strategy does not have a certain cushioning effect, it will be harmful to the motor. Therefore, it is necessary to test and analyse the control effect of pure position control and impedance control.

**Figure 13:**
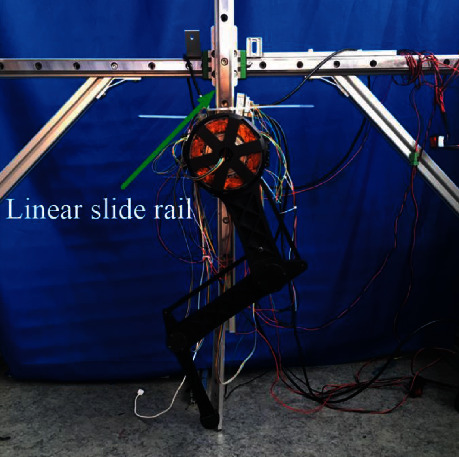
Diagram of free dropping apparatus of the single-leg prototype.

As shown in Figure 13, the experimental procedure is to set a fixed desired position through the impedance control and pure position control in the single-leg prototype in the suspended state (here, the desired force in the impedance control is set to 0). Then, at a certain height, the single-leg prototype was dropped freely, and the current and position change of the single-leg prototype motor were observed.

It should be noted that the drop height under the impedance control is 44 mm. Therefore, in the pure position control, the drop height was also set to 44 mm at first, but it is impossible to collect appropriate experimental data with high impact. At last, the drop height was determined to be 10 mm. In addition, through the data measurement and analysis of the two joint motors, the variation of current and position values of the hip motor during the falling process is not particularly obvious. Therefore, we did not collect the data on hip joint motor, but only current and position acquisition of the knee motor.

**Figure 14:**
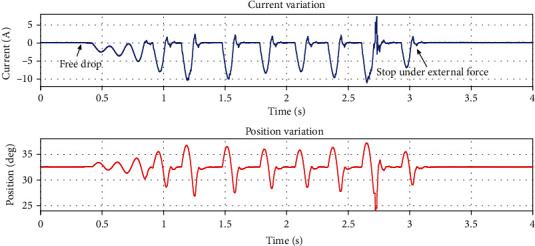
The current and position variation of the knee motor under pure position control.

By analyzing the experimental data in Figure 14, we can know that in the pure position control, even if it drops from a height of 10 mm, the position and current value of the motor show a tendency to disperse, that is, an unstable state. And the peak current during the whole process is 11.03 A as shown in Table 2. In the final stage, to avoid serious damage to the motor and the outside world, it is necessary to add external force to force the single-leg prototype to stop beating.

**Figure 15:**
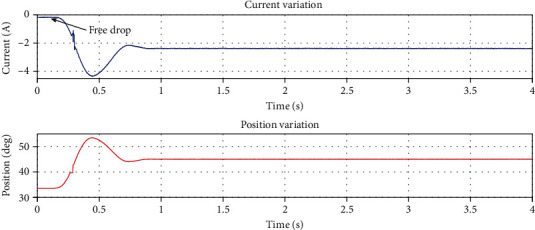
The current and position variation of the knee motor under impedance control.

In the impedance control as shown in Figure 15, we can see that although the drop height is 44 mm, which is 4.4 times in the above example, the peak current during the whole drop process is only 4.309 A as shown in Table 2, which effectively solves the impact on the motor during the dropping process.

### 3.4. Ground Compliance Identification

Through the previous analysis, we set up the corresponding experiments to identify the stiffness of the ground of various materials.

**Figure 16:**
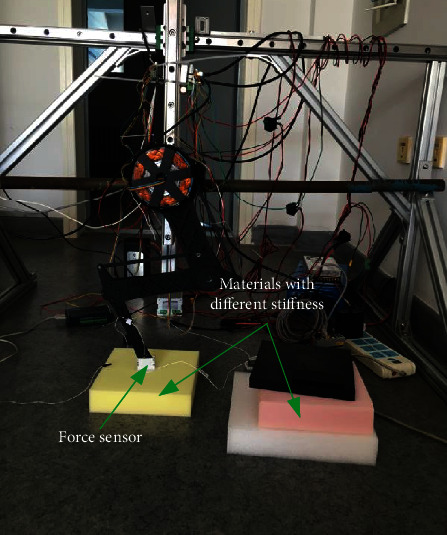
Diagram of the identification of various ground materials.

A portion of the experimental setup is similar to the previous motion control experiment where the motion capture system is used to capture the depth of the foot into the material. Since the stiffness is defined as the force acting on the unit displacement, it is also necessary to place a force sensor as shown in Figure 16 at the foot-end to measure the ground reaction force when the foot-end is in contact with the ground.

The experimental procedure consists of designing a control program that allows the foot-end point of the single-leg prototype to move vertically downward from above the ground material and penetrate the material, and the NDI Optotrak Certus and the force sensor collect the displacement and force information of the foot-end in real-time. Then, by observing the data of the collected foot-end force, we can judge the moment when the foot-end is in contact with the material, and then based on the displacement information at that moment, the penetration value data of the foot-end is obtained. With the penetration value and the foot-end force information, the stiffness of various ground materials can be identified by the recursive least squares method.

**Figure 17:**
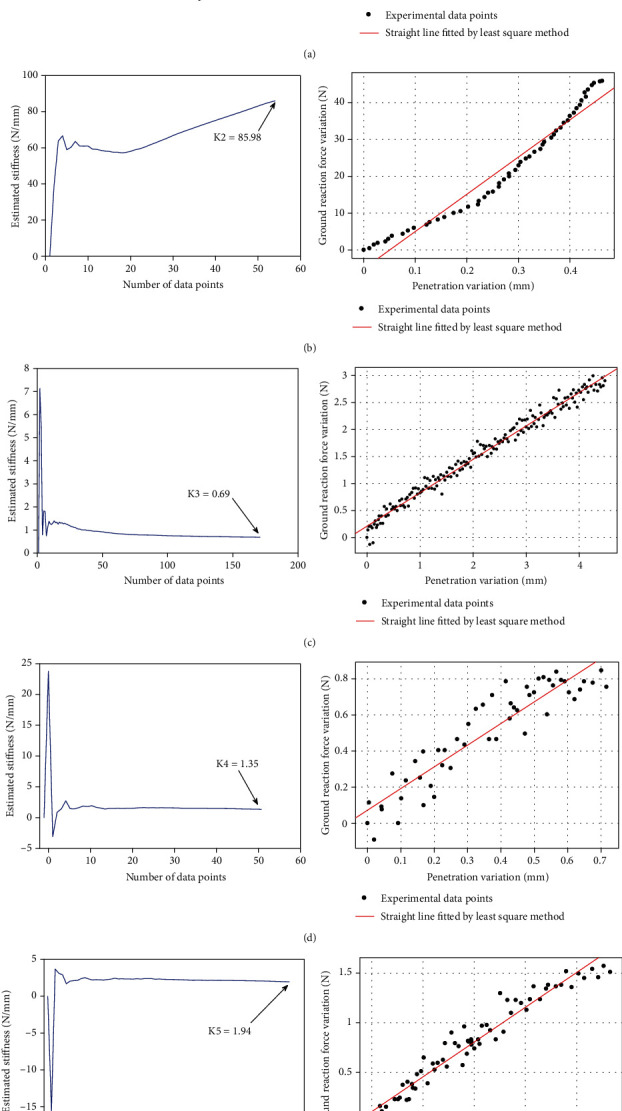
The recursive change process of stiffness and the fitting straight line. (a) Medium hard rubber. (b) High durometer rubber. (c) Low hardness sponge. (d) Medium hardness sponge. (e) High hardness sponge.

In the experiment, we selected five different materials, namely, material 1 is medium hardness rubber, material 2 is reinforced rubber, material 3 is low hardness sponge, material 4 is medium hardness sponge, and material 5 is high hardness sponge. The stiffness of the above five materials was obtained by the above experimental method, as shown in Figure 17.

## 4. Conclusion

Locomotion adaption for quadruped robots with ground compliance estimation is important, especially for high-speed locomotion. In this paper, the Cartesian coordinates of the foot-end are obtained through trajectory planning, and then the virtual polar coordinates in the impedance control are obtained through geometric transformation. The deviation from the planned and actual virtual polar coordinates and the expected force recognized by the ground identification system is sent to the impedance controller. The desired foot-end force is obtained and then the joint torque is obtained by the change of the Jacobian matrix and sent to the robot system. The status of the robot system is also fed back to the previous process. At last, several experiments are carried out for evaluating the performance including the ground compliance identification, the motion control and the comparison between pure position control and impedance control. In the future, the prosed control framework will be used on our quadruped robots for testing the robust when traversing on a wide variety of terrains.

## Figures and Tables

**Table 1:** The twelve control points of the Bézier curve.

	*x* (mm)	*y* (mm)
*c*_0_	-170	460
*c*_1_	-280.5	460
*c*_2_	-300	361.1
*c*_3_	-300	361.1
*c*_4_	-300	361.1
*c*_5_	0	361.1
*c*_6_	0	361.1
*c*_7_	0	321.4
*c*_8_	303.2	321.4
*c*_9_	303.2	321.4
*c*_10_	282.6	460
*c*_11_	170	460

**Table 2:** Comparison between pure position control and impedance control.

Control mode	Stability	Position steady-state error (deg)	Peak current (A)	Compliance
Impedance control	Stable	11.4	4.309	Soft
Position control	Unstable	—	11.03	Hard

## Data Availability

All data were experimentally obtained.

## References

[1] Hwangbo J., Lee J., Dosovitskiy A. (2019). Learning agile and dynamic motor skills for legged robots. *Science Robotics*.

[2] Orsolino R., Focchi M., Caron S. (2020). Feasible region: an actuation-aware extension of the support region. *IEEE Transactions on Robotics*.

[3] Ding L., Gao H., Deng Z. (2013). Foot-terrain interaction mechanics for legged robots: Modeling and experimental validation. *The International Journal of Robotics Research*.

[4] Wensing P. M., Wang A., Seok S., Otten D., Lang J., Kim S. (2017). Proprioceptive actuator design in the MIT cheetah: impact mitigation and high-bandwidth physical interaction for dynamic legged robots. *IEEE Transactions on Robotics*.

[5] Zeng X., Zhang S., Zhang H., Li X., Zhou H., Fu Y. (2019). Leg trajectory planning for quadruped robots with high-speed trot gait. *Applied Sciences*.

[6] Hutter M., Gehring C., Jud D. ANYmal - a highly mobile and dynamic quadrupedal robot.

[7] Li X., Zhou H., Feng H., Zhang S., Fu Y. Design and experiments of a novel hydraulic wheel-legged robot (WLR).

[8] Li X., Zhou H., Zhang S., Feng H., Fu Y. WLR-II, a hose-less hydraulic wheel-legged robot.

[9] Raibert M., Blankespoor K., Nelson G., Playter R. (2008). Bigdog, the rough-terrain quadruped robot. *IFAC Proceedings Volumes*.

[10] Seok S., Wang A., Otten D., Kim S. Actuator design for high force proprioceptive control in fast legged locomotion.

[11] Hu N., Li S., Huang D., Gao F. (2014). Crawling gait planning for a quadruped robot with high payload walking on irregular terrain. *IFAC Proceedings Volumes*.

[12] Semini C., Barasuol V., Goldsmith J. (2017). Design of the hydraulically actuated, torque-controlled quadruped robot HyQ2Max. *IEEE/ASME Transactions on Mechatronics*.

[13] Jung S., Hsia T. C., Bonitz R. G. (2004). Force tracking impedance control of robot manipulators under unknown environment. *IEEE Transactions on Control Systems Technology*.

[14] Kim D., Jorgensen S., Lee J., Ahn J., Luo J., Sentis L. (2019). Dynamic locomotion for passive-ankle biped robots and humanoids using while-body locomotion control. https://arxiv.org/abs/1901.08100.

[15] Neunert M., Stauble M., Giftthaler M. (2018). Whole-body nonlinear model predictive control through Contacts for quadrupeds. *IEEE Robotics and Automation Letters*.

[16] Bosworth W., Whitney J., Kim S., Hogan N. Robot locomotion on hard and soft ground: measuring stability and ground properties in-situ.

[17] Lee Y. H., Lee Y. H., Lee H. Trajectory design and control of quadruped robot for trotting over obstacles.

[18] Haberland M., Karssen J. G. D., Kim S., Wisse M. The effect of swing leg retraction on running energy efficiency.

